# The Process of Binding and Releasing of Genetic Material from Lipoplexes Based on Trimeric Surfactants and Phospholipids

**DOI:** 10.3390/ijms22147744

**Published:** 2021-07-20

**Authors:** Żaneta Polańska, Zuzanna Pietralik-Molińska, Daria Wojciechowska, Augustyn Moliński, Marek Weiss, Andrzej Skrzypczak, Maciej Kozak

**Affiliations:** 1 Department of Macromolecular Physics, Faculty of Physics, Adam Mickiewicz University, 61-614 Poznań, Poland; zan.ka@amu.edu.pl (Ż.P.); zuzannap@amu.edu.pl (Z.P.-M.); grobys.d@amu.edu.pl (D.W.); augmol@amu.edu.pl (A.M.); 2 NanoBioMedical Centre, Adam Mickiewicz University, Wszechnicy Pastowskiej 3, 61-614 Poznań, Poland; 3 Faculty of Materials Engineering and Technical Physics, Institute of Physics, Poznań University of Technology, Piotrowo 3, 60-965 Poznań, Poland; marek.weiss@put.poznan.pl; 4 Faculty of Chemical Technology, Poznań University of Technology, Berdychowo 4, 60-965 Poznań, Poland; andrzej.skrzypczak@put.poznan.pl

**Keywords:** tricationic surfactants, cytotoxicity, nucleic acids delivery, phospholipids, lipoplex

## Abstract

Nonviral vectors for gene therapy such as lipoplexes are characterized by low toxicity, high biocompatibility, and good transfection efficiency. Specifically, lipoplexes based on polymeric surfactants and phospholipids have great potential as gene carriers due to the increased ability to bind genetic material (multiplied positive electric charge) while lowering undesirable effects (the presence of lipids makes the system more like natural membranes). This study aimed to test the ability to bind and release genetic material by lipoplexes based on trimeric surfactants and lipid formulations of different compositions and to characterize formed complexes by circular dichroism (CD) spectroscopy and atomic force microscopy (AFM). The cytotoxicity of studied lipoplexes was tested on HeLa cells by the MTT cell viability assay and the dye exclusion test (trypan blue). The presence of lipids in the system lowered the surfactant concentration required for complexation (higher efficiency) and reduced the cytotoxicity of lipoplexes. Surfactant/lipids/DNA complexes were more stable than surfactant/DNA complexes. Surfactant molecules induced the genetic material condensation, but the presence of lipids significantly intensified this process. Systems based on trimeric surfactants and lipid formulations, particularly TRI_N and TRI_IMI systems, could be used as delivery carrier, and have proven to be highly effective, nontoxic, and universal for DNA of various lengths.

## 1. Introduction

The development of gene therapy methods is closely related to the search for effective and safe drug carriers capable of binding and transferring genetic material. Initially, a number of carriers based on viral capsids were mainly used (for review, see: [1,2]); however, now nonviral systems based on molecules capable of self-association and stable binding of nucleic acids, are also being tested [3]. The natural choice for the creation of such systems is lipids, the essential components of biological membranes, such as phosphatidylcholine or phosphatidylethanolamine derivatives, which bind genetic material to form stable complexes (lipoplexes) [4,5]. Such vehicles can transfer genetic material via cell membranes. Unfortunately, lipoplexes based on synthetic, cationic lipids, e.g., dioleoyl-3-trimethylammonium propane (DOTPA), dioctadecylamidoglycyl-spermine (DOSPA) or dioctadecylamidoglycylspermine (DOGS) are characterized by low transfection efficiency and may induce cytotoxic effects. To eliminate these defects, neutral lipids are used as an additive, e.g., dioleoylphosphatidylethanolamine (DOPE), dioleoylphosphatidylcholine (DOPC), or cholesterol. Due to the similar chemical structure (polar head and hydrophobic tail) and properties (ability to self-assembly), cationic surfactants may be a promising alternative to cationic lipids. Cationic surfactants should improve the efficiency while maintaining other positive features of lipids.

In the last two decades, the possibility of using several oligocationic surfactants as components in the formation of carriers for gene therapy has been demonstrated [6,7,8]. The architecture of dicationic surfactants is very alterable through synthesis that enables achieving the desired chemical properties [9]; therefore, a wide spectrum of dicationic surfactants has been proposed, with polar parts composed of various functional groups (pyridinium, imidazolium, histidine, etc.) [10,11,12,13,14].

In our research to date, we have focused mainly on the use of dicationic surfactants to create stable and nontoxic lipoplexes with nucleic acids, both siRNA oligomers and long and short DNA fragments [12,15,16], also including surfactants with amino acids molecules (glycine) as counter ions [17].

Our present studies focus on three trimeric surfactants, which differ in polar head groups (ammonium—N, imidazolium—IMI, and benzimidazolium—BEN). Our goals were to estimate their ability to bind with DNA, to evaluate the influence of natural lipids added to mixtures on complexation, and to characterize formed complexes. This study was performed on mixed systems composed of three types of trimeric surfactant (TRI_N, TRI_IMI, and TRI_BEN) and three lipids (dimyristoylphosphatidylcholine—DMPC, dioleoylphosphatidylethanolamine—DOPE, and dipalmitoylphosphatidylcholine—DPPC) in various composition and concentrations. Then, the interactions with nucleic acids were tested on three types of DNA varying in size, i.e., 21 bp, 185 bp, and 20 kbp.

## 2. Results

### 2.1. Binding and Releasing of Nucleic Acid

**Figure 1:**
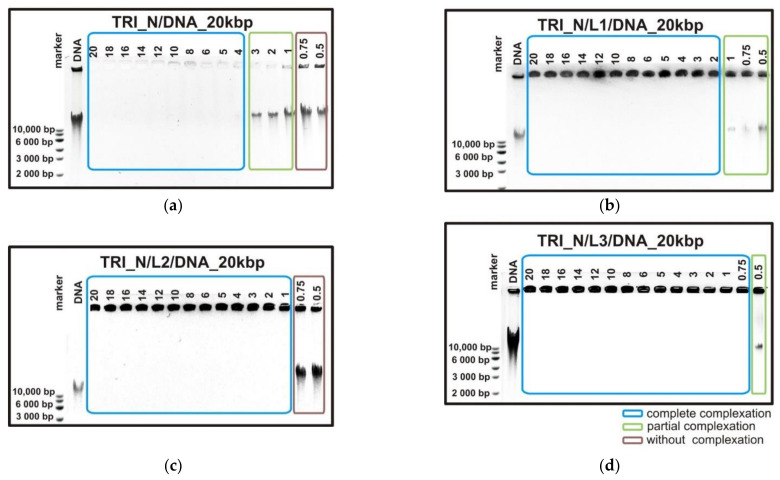
Agarose gel electrophoresis results obtained for lipoplexes based on high-molecular-weight DNA consisting of 20 kbp and (**a**) TRI_N, (**b**) TRI_N/L1, (**c**) TRI_N/L2, and (**d**) TRI_N/L3 systems.

Electrophoretic mobility tests were the primary method used to characterize and evaluate the complexes based on trimeric surfactants and nucleic acid. This technique allows determining the ability of studied compounds to complex with DNA. The exemplary results obtained for TRI_N surfactant and high-molecular-weight DNA are presented in Figure 1. The undisturbed migration of long DNA (20 kbp) results in a smeared band indicating that the sample is heterogeneous. For p/n > 4 (positive to negative charge ratio, see Section 4.2) marked with blue frame (Figure 1a), TRI_N surfactant forms stable complexes with DNA, and the net charge of studied systems is either zero or turns positive. This results in the inhibition of the DNA migration or migration of the entire complex in the opposite direction. In the case of lower surfactant concentration (p/n = 3, 2, 1), a residual DNA band is visible, but it is characterized by lower intensity and size relative to the reference DNA, indicating that some portion of nucleic acid remained unbound. When a surfactant–DNA complex is not formed, one can observe unrestricted migration of the sample as can be seen for the p/n ratios of 0.75 and 0.5 (marked with red frame). Therefore, the efficiency of binding the nucleic acid can be characterized by the lowest p/n value required to obtain the complete complex formation. In the case of the pure solution of surfactant TRI_N, it was achieved for p/n = 4.

The addition of phospholipids to the systems influenced the complexing process and its efficiency significantly. When beside the TRI_N surfactant, the complex is based on one type of phospholipid molecule (DMPC—L1) (Figure 1b), and the p/n value required to bind the entire portion of DNA decreases to 2. In the case of the two-component phospholipid mixture (DMPC/DOPE—L2) (Figure 1c), the required p/n value equals 1, and with an increasing amount of phospholipid components (DMPC/DPPC/DOPE—L3) (Figure 1d), it decreases even further, reaching value p/n = 0.75. Thus, one can conclude that the concentration of surfactant necessary to form the complexes with DNA can be reduced by addition of phospholipid molecules. In addition, the diversity of the lipids has increased the complexation efficiency, such that the more varied the mixture, the lower the concentration of surfactant required. Additionally, with phospholipids mixtures, the migration in the opposite direction is blocked implying that the positive charge of surfactant molecules is not exposed.

**Figure 2:**
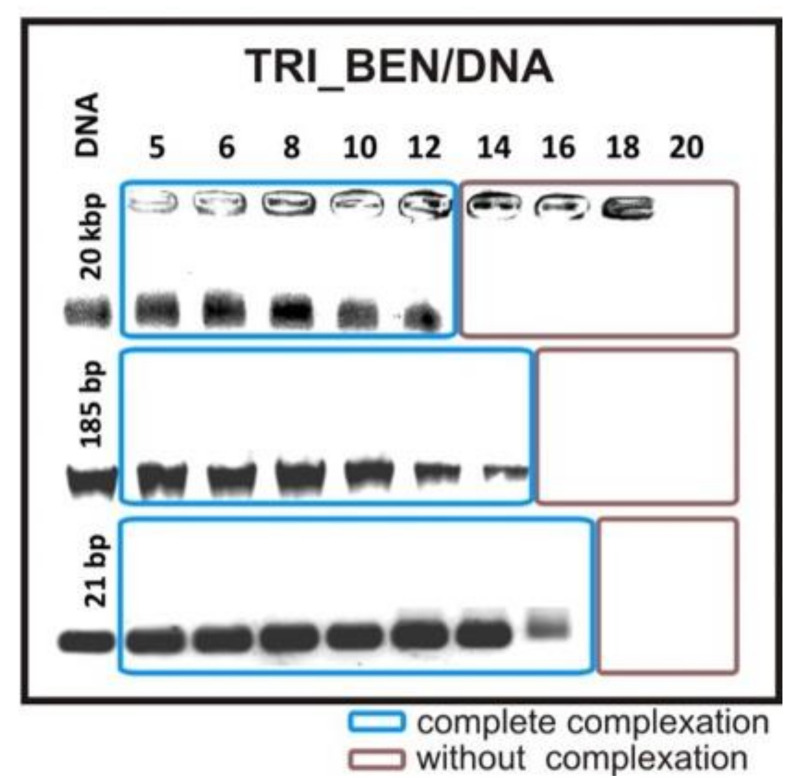
Agarose gel electrophoresis results obtained for lipoplexes based on TRI_BEN surfactant and all types of studied DNA molecules.

The results obtained for all studied systems are summarized in Table 1. Several factors influencing the binding ability of lipoplex can be determined, most of all, the type of oligomeric surfactant used. For all studied nucleic acids, surfactant TRI_BEN, built of the largest and stiffest head groups, was the least efficient. Successful complex formation was reached for the systems with TRI_BEN surfactant for p/n = 14 with 20 kbp DNA, for p/n = 16 with smaller 185 kbp DNA, and the smallest DNA fragment −21 bp for p/n = 18. The highest efficiency was achieved by surfactant TRI_N with high-molecular-weight DNA—p/n = 4 and smaller DNA fragment—p/n = 4, and was as efficient as surfactant TRI_IMI for binding small DNA oligomer—p/n = 8. One can also notice that the type of nucleic acid influences the attained efficacy, i.e., the smaller the DNA molecule, the higher the concentration is required to form stable complexes. This tendency is illustrated by electrophoretic separation for systems based on TRI_BEN and tested DNAs, presented in Figure 2. It may be connected to the degree of condensation of longer nucleic acid molecules and adopted conformation.

As can be seen in Table 1, for all studied systems, the presence of phospholipids reduced the p/n ratios required for complexation significantly and, hence, the concentration of trimeric surfactants. The influence of phospholipid composition is not well established at this point in the study. For the described systems based on TRI_N with 20 kbp DNA (Figure 1), the correlation was simple, i.e., the efficiency followed the diversity of lipids composition. For the analogous case of TRI_IMI and TRI_BEN, the best results were obtained in the case of two-component system—L2. In addition, for small DNA oligomer (21 bp), mixture L2 in combination with TRI_N gave slightly better results, whereas there were no differences between lipids composition and efficiency in the case of other surfactants. To establish reliable correlations, investigation of a more diverse group of lipid mixtures is required.

**Figure 3:**
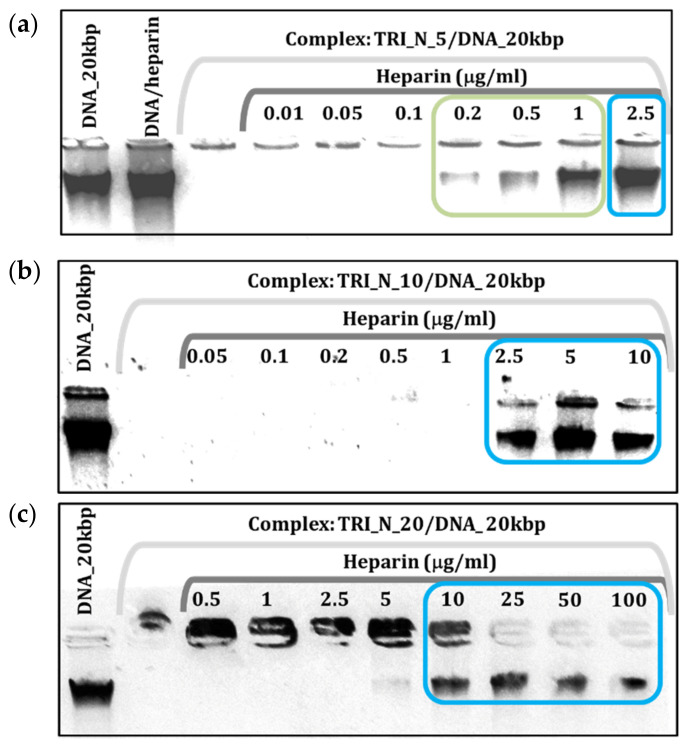
Agarose gel electrophoresis results obtained for lipoplexes based on (**a**) TRI_N/DNA_20kbp for p/n = 5, (**b**) for p/n = 10, and (**c**) for p/n = 20 with increasing heparin concentration.

The release of the DNA from the complex is discussed on the example of TRI_N/DNA_20kbp for p/n = 5 (Figure 3a). The first sample in this image is the reference DNA solution, followed by the same solution incubated with heparin at a concentration of 2.5 µg/mL to exclude possible heparin–DNA interactions. The next sample is another reference, i.e., complex formed by TRI_N/DNA_20kbp for p/n = 5, which based on the binding tests does not migrate in the electric field. Subsequent samples contain the same complex but are incubated with an increasing concentration of heparin. For the concentration of 0.2 µg/mL of heparin, a slight streak appears, indicating a partial release of DNA. Increasing the concentration of heparin, DNA is being completely released from the complex for the concentration of 2.5 µg/mL. For higher surfactant concentration, p/n = 10 (Figure 3b), the same amount of heparin caused the release of nucleic acid, while for doubled surfactant concertation, p/n = 20 (Figure 3c), the required heparin concentration was four times higher. This type of analysis can be used to qualitatively compare the tested system and assess the stability of formed complexes.

**Figure 4:**
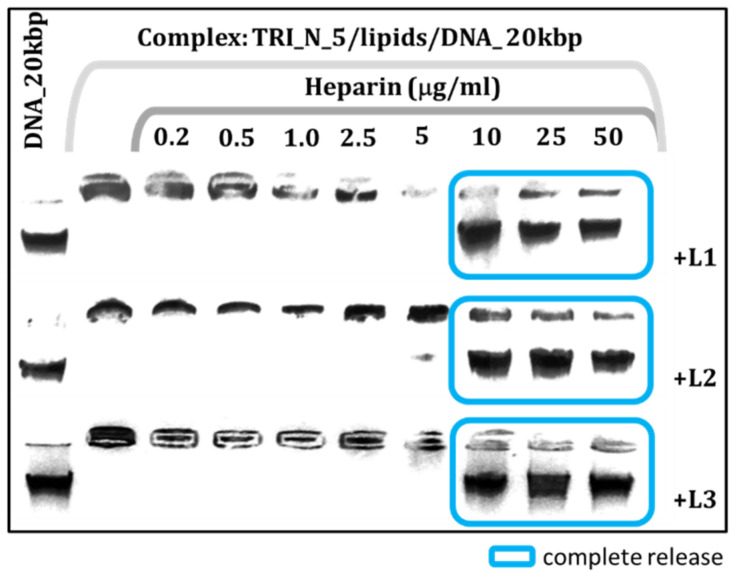
Agarose gel electrophoresis results obtained for lipoplexes based on TRI_N/lipids/DNA_20kbp with increasing heparin concentration.

The addition of phospholipids to the systems (Figure 4) yielded more durable and stable carriers as the concentration of heparin needed to uncage the DNA; therefore, it migrates freely during electrophoretic separation and is a few fold higher (10–25 µg/mL) than for corresponding samples without lipids. Results obtained for all studied systems are gathered in Table 2. The weakest complexes were formed by the TRI_BEN surfactant-based systems, which were also the least efficient at binding nucleic acid, and the most durable TRI_N- and TRI_IMI-based system combined with lipids.

### 2.2. The Conformation of Nucleic Acid

**Figure 5:**
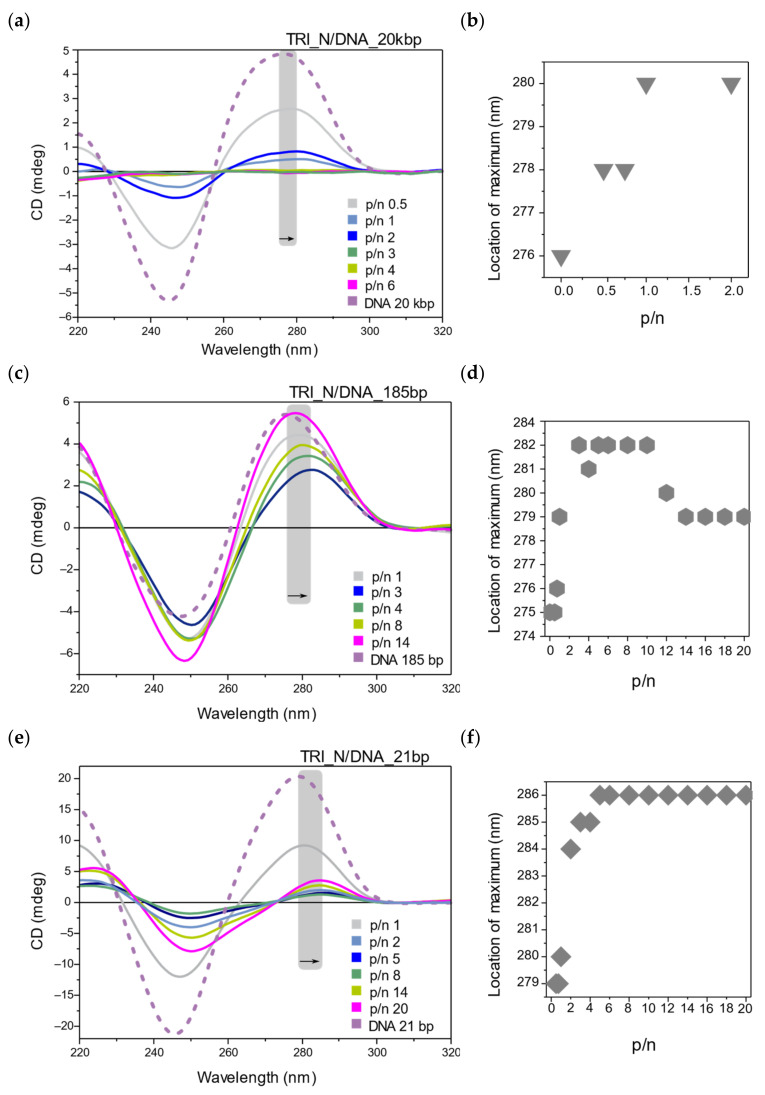
Circular dichroism studies of TRI_N surfactant with different DNAs. (**a**) CD spectra collected for TRI_N/DNA_20kbp systems and (**b**) corresponding shifts of positive maximum with increasing p/n ratio. (**c**) CD spectra collected for TRI_N/DNA_185bp systems and (**d**) corresponding shifts of positive maximum with increasing p/n ratio. (**e**) CD spectra collected for TRI_N/DNA_21bp systems and (**f**) corresponding shifts of positive maximum with increasing p/n ratio.

The conformation of all DNA samples (21 bp, 185 bp, and 20 kbp), selected as model nucleic acids, were first studied in a solution (5 mM sodium phosphate buffer, pH 6.8) without surfactants. The circular dichroism (CD) spectra for studied DNA solutions and mixtures of DNA with TRI_N surfactant are presented in Figure 5a–f. All DNA samples exhibited B-DNA conformation manifested by characteristic bands in the CD spectrum: minimum at about 245 nm in the negative band, maximum at about 276 nm in the positive band, and intersection with the *X*-axis at about 260 nm.

For the surfactant–DNA systems, any unequivocal trend of changes observed in CD spectra, and thus dramatic changes of DNA structural phases, was not noted. The main effects observed were changes in the intensity of the characteristic bands and the redshift of their position.

The CD spectra for the TRI_N surfactant and 20 kbp DNA systems (Figure 5a), with increasing surfactant concentration (p/n values), were characterized by a gradual decline of the intensity and a complete flattening of the CD spectrum from p/n = 3. For the system characterized by p/n = 1, the CD spectrum reached an intensity equal to 20% of the intensity of the reference DNA spectrum, while for p/n = 2, this value decreased to 10%. A shift of the positive band maximum by 4 nm toward longer wavelengths was also observed (Figure 5b).

For low p/n charge ratio (0.5 and 0.75) in mixed systems of TRI_N surfactant with 185 bp DNA (Figure 5c), the intensity of CD spectra decreased proportionally both for the positive and negative bands. However, from the p/n = 1, a decrease of 30% in the intensity of the positive band was observed, but the intensity of the negative band increased by 10% in relation to the intensity of the reference DNA spectrum. The lowest intensity of the positive CD band was noted for p/n = 3 and declined to ~40% of the reference intensity value, and the maximum of the positive band was shifted to 282 nm (Figure 5d). From p/n = 4, the intensity of the positive band gradually increased again, reaching a value close to the reference DNA positive band intensity for the highest concentrations of TRI_N surfactant. The greatest increase in the intensity of the negative band (by 60%) was observed for the system characterized by p/n = 14.

Similar effects were observed for the TRI_N/DNA_21bp systems (Figure 5e). For lower p/n values (0.5–1), the intensity of the CD spectrum decreased by approximately 50% in the comparison to the reference spectrum for DNA; however, the position of the positive band did not change. From p/n > 2, there was a significant flattening of the CD spectrum and the redshift of its positive band. The maximum flattening of the spectrum in relation to the reference DNA spectrum was observed at p/n = 8. For systems characterized by p/n > 8, the intensity of the spectrum increased again but did not reach the intensity of the reference DNA spectrum. The maximum of the positive band shifted to 286 nm (by + 7 nm) (Figure 5f). Moreover, an isodichroic point was observed at 273 nm.

**Figure 6:**
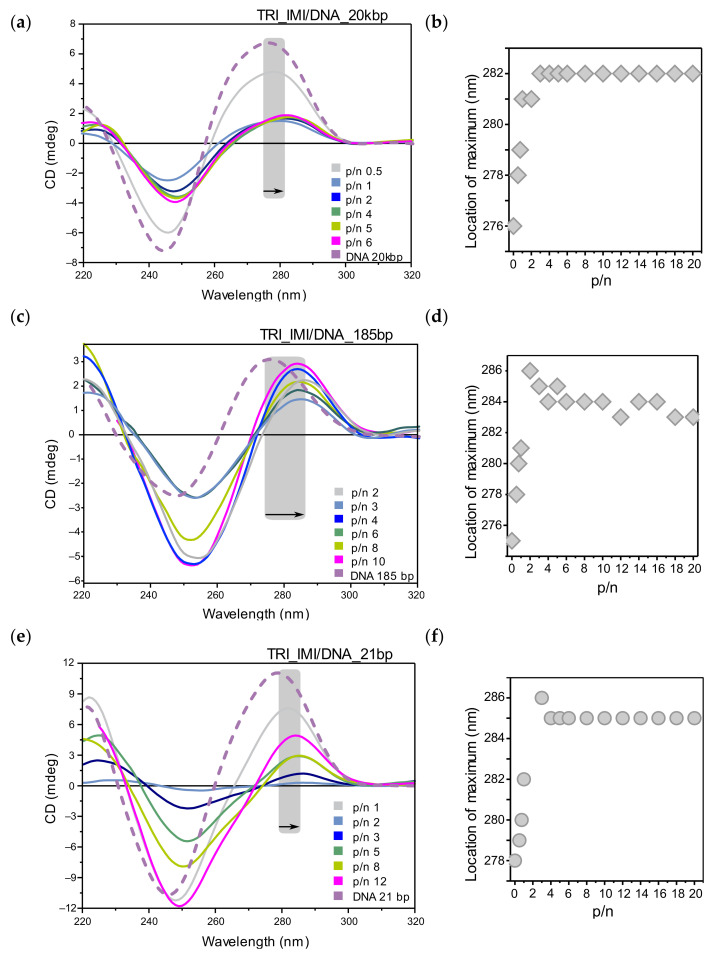
Circular dichroism studies of TRI_IMI surfactant with different DNAs. (**a**) CD spectra collected for TRI_IMI/DNA_20kbp systems and (**b**) corresponding shifts of positive maximum with increasing p/n ratio. (**c**) CD spectra collected for TRI_IMI/DNA_185bp systems and (**d**) corresponding shifts of positive maximum with increasing p/n ratio. (**e**) CD spectra collected for TRI_IMI/DNA_21bp systems and (**f**) corresponding shifts of positive maximum with increasing p/n ratio.

The circular dichroism spectra for the mixed system TRI_IMI surfactant and 20 kbp DNA are presented in Figure 6a. For low p/n values (0.5, 0.75), the CD spectrum intensity slightly decreases in comparison to the reference DNA spectrum. From the charge ratio p/n = 1, the CD spectrum intensity significantly decreases, and the maximum of the positive band is shifted toward longer wavelengths (Figure 6b), reaching the value of 282 nm (shift by 6 nm, p/n > 3). For the system at p/n = 6, an increase in the intensity of the negative band was also observed.

For the next studied system, the mixture of TRI_IMI surfactant and DNA 185 bp long (Figure 6c), we observed a decrease of the intensity of the negative band in CD spectrum in relation to the reference DNA spectrum, also accompanied by a slight decrease in the intensity of the positive band. This effect was most pronounced in the CD spectrum of lipoplex at p/n = 4. Then, the intensity of the negative band decreases (p/n = 6) to grow back for systems at p/n > 8. The position of the maximum of the positive band for charge ratio p/n > 2 shifted toward higher wavelengths (up to 286 nm) (Figure 6d). With increasing surfactant concentration, one can observe redshift of positive band and the intersection point with increased intensity of the negative band indicating strong chiral DNA condensation in the so-called *Ψ*-DNA form [18,19]. Therefore, in these systems, DNA condensation (formation of *Ψ*-DNA form) is likely due to interaction with the TRI_IMI surfactant.

For systems based on DNA oligomers with a length of 21 bp and TRI_IMI surfactant (Figure 6e), only a slight decrease in spectral intensity was observed for systems with p/n < 1; however, for p/n = 2, significant flattening of the CD spectrum was visible. By increasing the surfactant concentration (p/n > 3), the negative band grew more than the positive band but did not exceed the intensity of the negative band of the reference DNA spectrum. While for the system at charge ratio p/n > 12, the negative band exhibited a greater intensity than for DNA. The maximum of the positive band in the spectra of lipoplexes at charge ratio p/n = 4 (and higher) was shifted to 286 nm (the shift by 8 nm). This general behavior is in good agreement with previously tested by our TRI_IMI reference systems with DNA 21 bp, prepared in the lower DNA concentrations range [20].

**Figure 7:**
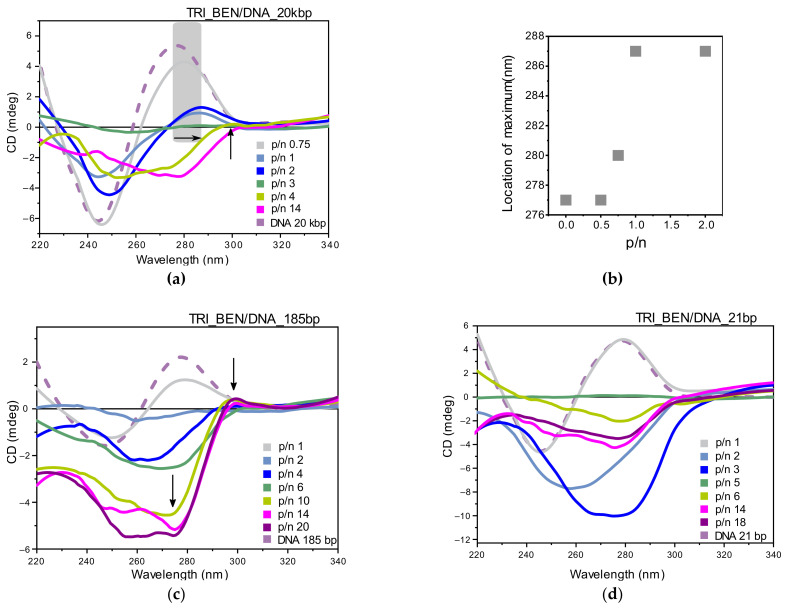
Circular dichroism studies of TRI_BEN surfactant with different DNAs. (**a**) CD spectra collected for TRI_BEN/DNA_20kbp systems and (**b**) corresponding shifts of positive maximum with increasing p/n ratio. (**c**) CD spectra collected for TRI_BEN/DNA_185bp systems and (**d**) CD spectra collected for TRI_BEN/DNA_21bp systems.

The circular dichroism spectra collected for the 20 kbp DNA system with the TRI_BEN surfactant are presented in Figure 7a. For systems at p/n range 1–2, the spectrum intensity decreased; however, the shape of the spectrum characteristic for the B-DNA form was maintained. The positive band maximum shifted toward longer wavelengths, reaching 287 nm (Figure 7b). For TRI_BEN/DNA_20kbp system, characterized by p/n = 3, the CD spectrum was completely flattened, while increasing surfactant concentration (p/n ≥ 4) induced regrowth of the CD signal. However, it was accompanied by significant change in the spectrum shape. The CD spectra for these systems exhibited two peaks: a strong negative band with a minimum at 253 nm and a slight positive band at 300 nm, which suggests the DNA condensation process. Gradually, for systems at p/n from 6 to 14, the minimum shifted reaching the location of 280 nm. It is worth noting that the results of the electrophoretic tests indicated the formation of a stable complex after reaching p/n = 14. Therefore, based on the CD results, we can conclude that in the TRI_BEN/DNA_20kbp systems from p/n = 4, surfactants were bound with the nucleic acid, condensing its structure, but complete complexation did not take place. Moreover, after the formation of the complex at p/n > 14, the formed structures were insoluble and sediment from the solution.

For systems based on TRI_BEN surfactant with 185 bp DNA (Figure 7c), a similar relationship was observed as for systems composed of DNA 20 kbp and TRI_BEN. The CD spectra for systems at low p/n values (0.5, 0.75, 1) were characterized by decreased intensity. For p/n = 2, the spectrum was completely flattened (while the sample was homogenous and clear), and the DNA in the sample with p/n = 3 was precipitated. For systems with charge ratio from p/n ≥ 4, the increase in the intensity of the negative band was observed, and its minimum was shifted from 265 nm (for p/n = 4) up to 275 nm (for p/n = 20). A slight positive maximum was also observed, and it shifted from 292 nm (for p/n = 4) to 299 nm (for p/n = 20). As for TRI_BEN/DNA_20kbp systems, also for samples with shorter −185 bp DNA, precipitation was observed at p/n > 14. These were conditions at which full complexation took place (based on electrophoresis).

Last studied systems based on short (21 bp) DNA oligomers (Figure 7d) mixed with TRI_BEN surfactant. CD spectra of systems at low surfactant concentration (p/n = 0.5, 0.75, 1) exhibited no change from the reference DNA spectrum. On the other hand, for systems at p/n = 2 and 3, a strong negative band with a minimum at 258 and 274 nm was observed and was characterized by the intensity of about –8 and −10 mdeg for p/n = 2 and 3, respectively. Samples with p/n = 4 and 5 were opaque, and the CD spectrum was flat. From p/n = 6, the CD spectrum had one negative band with a minimum at 278 nm, and its position was not shifted. However, the band intensity increased with the surfactant concentration in the system, reaching the lowest negative value for p/n = 14 (about −3.9 mdeg). Stable complexes based on TRI_BEN/DNA_21bp are formed for p/n ≥ 18 (confirmed by electrophoretic tests); however, even for lower charge ratio (p/n ≥ 6), DNA condensation occurs as a result of the interaction of DNA chains with surfactant molecules.

In summary, the TRI_BEN surfactant strongly interacts with DNA, condensing its structure (probably in the *Ψ*-DNA form) even before the full formation of stable and complete complexes. However, in most cases, after reaching the concentration required for full complexation, precipitation was observed.

### 2.3. The Cytotoxicity Tests

**Figure 8:**
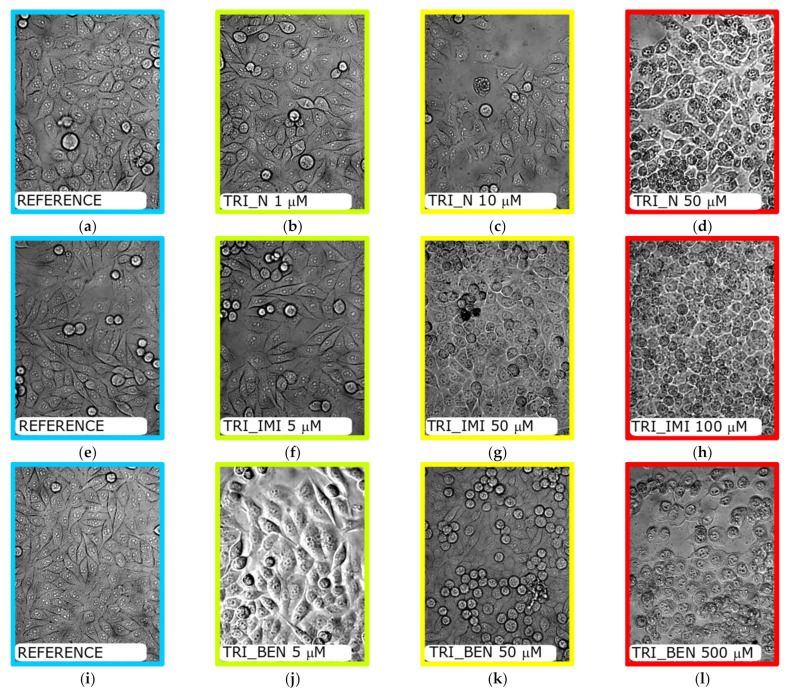
Morphological changes in the HeLa cells after incubation with surfactants at increasing concentrations. Untreated cells (**a**,**e**,**i**), (**b**–**d**) cells treated with TRI_N at concentrations 1, 10, and 50 μM; (**f**–**h**) cells treated with TRI_IMI at concentrations 5, 50, and 100 μM; (**j**–**l**) cells treated with TRI_BEN at concentrations 5, 50, and 100 μM.

**Figure 9:**
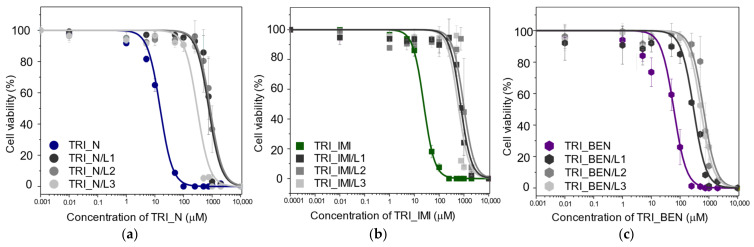
The results of the MTT assay after HeLa cells incubation with surfactants and surfactants and lipids mixtures (L1–L3) at increasing concentrations. (**a**) dose response curve showing of TRI_N, (**b**) TRI_IMI, and (**c**) of TRI_BEN based systems.

The typical image of HeLa cells is characterized by a spindle cell shape with a visible nucleus, adjacent to the bottom of the plate (Figure 8a,e,i). Addition of low-concentration of surfactants did not change their morphology (Figure 8b,f,j). However, increasing their concentration caused changes in the ratio between living and dead cells (see Figure 9 and Table 3). The (Figure 8c,g,k) images show that some cells lost their characteristic spindle shape and detached from the bottom of the plate when incubated with surfactant concentration close to *EC_50_*. When the concentration of surfactant reaches a critical level, inducing 100% cell death (Figure 8d,h,l), the cells became round shaped, exhibiting “overgrown” vesicular structures inside.

Cytotoxicity of the studied surfactants as well as surfactants and lipids mixtures was determined using the MTT cell viability assay (Figure 9), which is based on tetrazolium salt conversion to purple formazan by dehydrogenase enzymes occurring in the living cell. We observed that the cell viability decreases with increasing concentration of the tested substances. Among studied surfactants, TRI_N showed the highest cytotoxicity, while TRI_BEN is the least toxic (Table 3). Moreover, we observed that the *EC_50_* increases significantly by the addition of lipids. Especially the L2 lipid mixture exhibited high cytoprotective activity, causing the increase of *EC_50_* value, from 10 times for TRI_BEN surfactant to more than 50 times for TRI_N. The L1 mixture exhibited slightly lower effectivity than L2, while the L3 improves the cell viability at the lowest degree, however, still being from 5 times in the case of TRI_BEN surfactant to more than 20 times for TRI_IMI.

**Figure 10:**
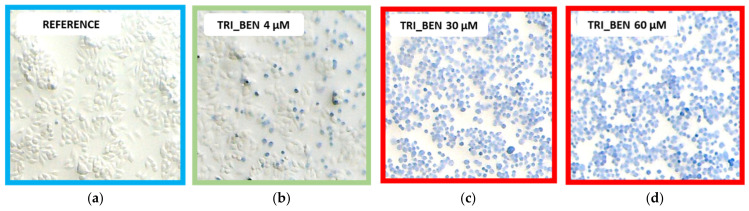
The results of the HeLa cells trypan blue staining after incubation with TRI_BEN. (**a**) Untreated cells and (**b**–**d**) with increasing concentrations of surfactant.

To confirm above-presented results we performed the trypan blue staining. This test is based upon the concept that impermeable dyes (such as trypan blue) cannot be taken up by viable cells contrarily to the dead once. Control cells have the typical morphology, i.e., a slight spindle shape, and are transparent (Figure 10a). In the presence of a low dose of surfactants, i.e., TRI_BEN (Figure 10b) numerous pale blue-stained, spherical shape cells appeared in the field of view. The blue color of cells intensified with increasing concentration of surfactant, as well as the number of spherical cells. Further increase of surfactant concentration (Figure 10c,d) resulted in deeper blue color of the cells.

**Figure 11:**
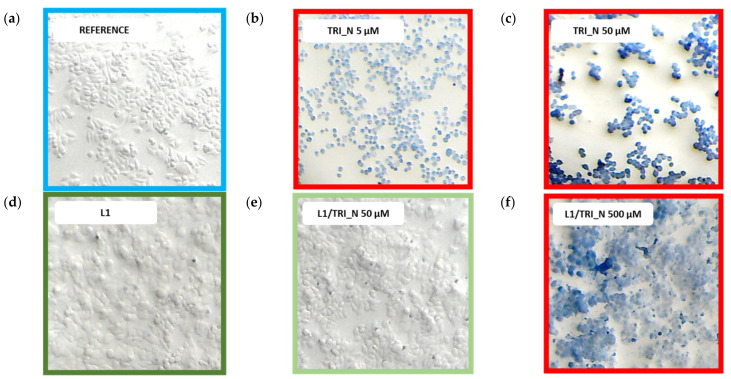
The results of the HeLa cells trypan blue staining after incubation with TRI_N. (**a**) Untreated cells, (**b**,**c**) samples with increasing concentrations of surfactant without L1, (**d**) cells treated with L1, and (**e**,**f**) samples with increasing concentrations of surfactant in the presence of L1.

Subsequently, we tested mixtures of surfactants and lipids, and the exemplary images are presented in Figure 11 for TRI_N surfactant. Control cells had typical morphology and were transparent (Figure 11a). The same one can be observed after exposing the cells to phospholipid solution L1 (Figure 11d). In the presence of low-dose TRI_N (5 μM), cells started to be more spherical and pale blue (Figure 11b), while the addition of L1 strongly suppressed this effect as can be seen for 10 times higher surfactant concentration in Figure 11e. For a corresponding sample with 50 μM TRI_N surfactant without lipid (dose above *EC_50_* based on the MTT assay), cells were dark blue and spherical, and some of them detached from the bottom of the plate (Figure 11c). The addition of L1 prevented these effects, and even at 500 μM TRI_N concentration (Figure 11f), L1 causes the cells to take up less trypan than at 50 μM. As summarized in Table 4, surfactants TRI_N and TRI_IMI presented similar toxicity while TRI_BEN was least toxic. In the studied concentration range, for all mixed surfactant/lipids systems, stained cells were visible at the same concentration value.

### 2.4. Topographic AFM Studies

**Figure 12:**
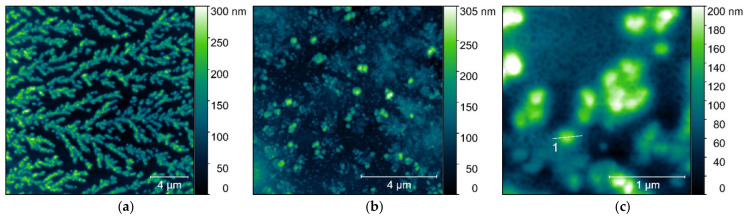
AFM images of (**a**) the high-molecular-weight DNA samples at 0.08 µM and (**b**,**c**) 0.02 µM concentration with marked cross-section, (**c**) for one of the visualized structures (see summarized cross-sections in Figure 13h).

**Figure 13:**
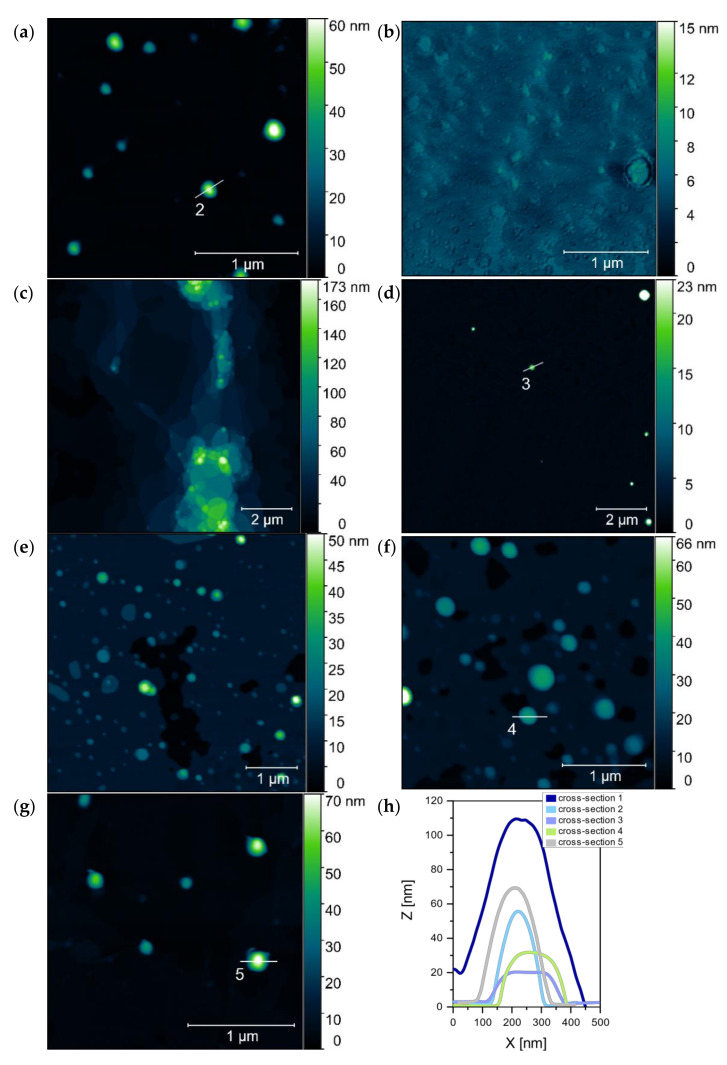
AFM images of the studied complexes based on TRI_N surfactant: (**a**) TRI_N/DNA_20kbp, p/n = 5 (**b**) TRI_N/L1/DNA_20kbp, p/n = 5 (**c**,**d**) TRI_N/L2/DNA_20kbp, p/n = 5 (**e**,**f**) TRI_N/L3/DNA_20kbp, p/n = 5 (**g**) and TRI_N/DNA_20kbp, p/n = 8 (**h**) together with a set of cross-sections highlighted on panels (**a**,**d**,**f**,**g**) and Figure 12c.

The atomic force microscopy (AFM) was used to visualize the microstructures of the most promising trimeric surfactant (TRI_N, TRI_IMI) complexes, also combined with the lipid formulations (L1, L2, L3) with the high molecular weight DNA (20 kbp). Additionally, the average height of formed structures was determined. The sample preparation method applied by us, and the procedure of AFM data collection limited the visualization of the reference samples—trimeric surfactants. The amphiphilic structures formed by these surfactants were unstable besides the solution. The reference DNA sample was prepared in a low ionic strength solution; therefore, it was present in a coiled form instead of separated DNA chains (Figure 12). These aggregates, during the sample drying, formed usually cylindrical or spherical structures induced by DNA condensation. The AFM image presented in Figure 12a shows DNA molecules (in high concentration—0.08 µM), deposited in a form of the aggregated structures and organized in branched patterns, which were formed during the drying of the sample. Similar microstructures were also observed for DNA at a lower concentration (0.02 µM), where many spherical DNA structures were visualized (Figure 12b). The size analysis of the observed microstructures was performed only for isolated objects (see the cross-sections marked in Figure 12c and summarized in Figure 13h). The average height of the microstructures was 93 ± 6 nm and was calculated from 31 individual cross-section measurements. Despite the DNA aggregates being the most common structure, the nonaggregated DNA form was also visible on the mica surface, between the spherical structures (Figure 12c).

The AFM analysis of the TRI_N/DNA_20kbp sample at p/n = 5, shown in Figure 13a, revealed some isolated, spherical objects with a height of 48 ± 4 nm (calculated from 97 cross-sections). The obtained value is almost two times smaller than the size determined for the reference DNA structures, indicating DNA condensation due to the interaction with the surfactant. For a corresponding sample with L1 lipid—TRI_N/L1/DNA_20kbp, p/n = 5 (Figure 13b)—it was not possible to measure the height, because of a tendency to form a number of large and irregular aggregated structures with barely distinguishable borders. TRI_N/L2/DNA_20kbp system (Figure 13c,d) formed large polydisperse cylindrical aggregates when deposited on mica surface. According to Figure 13c, the maximum height of these microstructures was estimated at 173 nm. However, some areas are covered by isolated, homogeneous-in-shape, spherical objects (Figure 13d). The mean height characterizing these objects was 26 ± 5 nm (calculated from 19 cross-section measurements). This result shows that the addition of the lipid molecules to the system promotes further DNA condensation. Such a process is probably supported by the hydrophobic interactions between lipid and the tricationic surfactant molecules, which leads to the strong molecular organization of the lipoplex structure.

However, despite the presence of the L3 lipids mixture in the TRI_N/L3/DNA_20kbp system at p/n = 5 (Figure 13e,f), such a strong DNA condensation was not observed. Here, the mean height of the observed complexes was 32 ± 4 nm, (calculated from 97 cross-section measurements). The topographical analysis showed numerous, spherical, and isolated objects with no tendency to aggregate into larger structures; a deposited lipid film was also visible. To support the initially posed hypothesis about the possible aggregation processes of the complexes for larger surfactant concentration, the TRI_N/DNA_20kbp system with p/n = 8 value was also subjected to the analysis (Figure 13g). It has been observed that the mean height of observed objects was 58 ± 4 nm (calculated from 111 cross-section measurements). This value was larger than for complex prepared for p/n = 5 value (48 ± 4 nm). Thus, one may say that by increasing the concentration of the surfactant, an additional molecular layer of surfactant molecules is probably added to the complex. In Figure 13h a set of cross-sections which were marked in Figure 12c and Figure 13a–g is presented.

**Figure 14:**
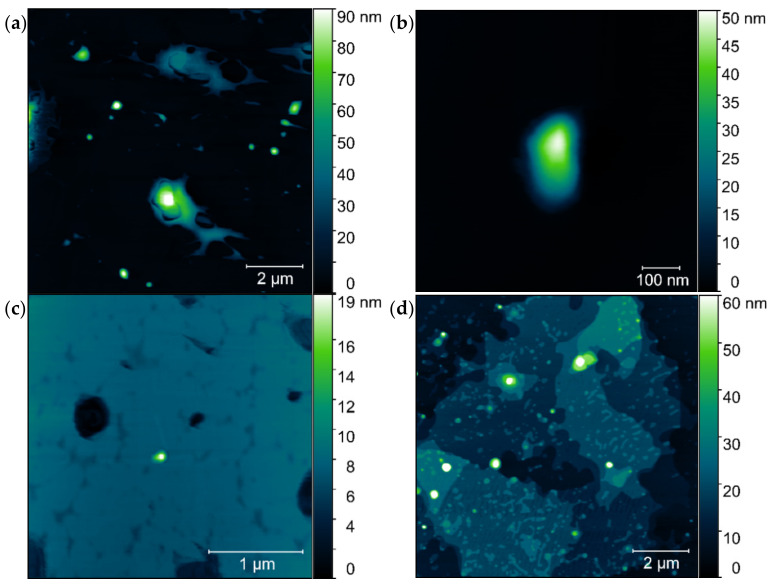
AFM images of the complexes based on TRI_IMI surfactant (**a**,**b**) TRI_IMI/DNA_20 kbp, p/n = 5; (**c**) TRI_IMI/L1/DNA_20 kbp, p/n = 5; and (**d**) TRI_IMI/L3/DNA_20 kb, p/n = 5.

Complexes based on TRI_IMI surfactant (p/n = 5) were also analyzed in terms of their topography. This surfactant also promoted the creation of stable complexes together with the DNA 20 kbp (Figure 14a,b). The shape of prepared complexes was ovoidal and irregular, contrary to the previously described systems (for comparison see Figure 13). During AFM experiments, it was also found that the adhesion to the mica substrate was relatively poor. The number of aggregates per unit area, as well as the height of these complexes, was notably lower than for TRI_N systems. The height of the studied systems (Figure 14) was: TRI_IMI/DNA_20kbp, p/n = 5–44 ± 9 nm (48 measurements); TRI_IMI/L1/DNA_20kbp, p/n = 5–20 ± 6 nm (18 measurements); and for TRI_IMI/L3/DNA_20kbp, p/n = 5–28 ± 3 nm (97 measurements).

AFM analysis showed that the size of the complexes is influenced by both: the type of the surfactant (TRI_N created larger complexes than TRI_IMI) as well as its concentration (the higher the concentration of the surfactant used, the larger the structures were found to be). Moreover, any addition of the lipids, depending on their composition, influences the size and shape of the complex formed. The complexes with lipids were smaller than complexes without them, while being more polydisperse. The DNA condensation degree is related to the size of the complex, and this can influence the ability of such a complex to be transfected to living cells.

## 3. Discussion

Lipid-based formulations have been proposed previously as an alternative to viral systems in the delivery of nucleic acids for gene therapy [21,22,23]. Recently, these systems have also been considered as delivery systems of novel vaccines based on mRNA [24,25]. A variety of lipid-based systems have been tested, and many promising microstructures were developed, such as liposomes [26] or lipid-based nanoparticles [27]. However, their long-term stability and transfection efficiency are rather limited [28,29]. Synthetic amphiphilic compounds from a group of dicationic (gemini-type) surfactants or higher oligomeric forms can effectively bind nucleic acids; however, they exhibit higher toxicity to living cells. Moreover, trimeric surfactants studied in this paper are characterized by higher cytotoxicity than dimeric surfactants already described by Pietralik et al. [17]. Therefore, combining selected trimeric surfactants with phospholipids can reduce the toxicity of the surfactants, while maintaining good complexity of the genetic material.

The ability to form stable complexes with nucleic acids by the studied trimeric surfactant–lipid systems is a basic feature that should be used for screening and selection of the most promising formulations that can be used further for transfection tests. In our studies, we selected these systems based on electrophoretic tests, discussed in Section 2.1. The general rule observed was that the presence of lipids in complexes with tested surfactants efficiently improved the DNA complexation at lower surfactant concentrations. The dependence of the surfactant concentration required for lipoplex formation in surfactant/DNA and surfactant/lipid/DNA systems with the length of the complexed nucleic acid was also observed. Earlier studies by Muñoz-Úbeda et al. [30] showed that the plasmid DNA in comparison to linear DNA requires a lower surfactant concentration for the complexation, due to the supercoiled structure of plasmid, thus reducing surface area and consequently lowering the effective negative charge. The abovementioned effect is also present in our studies but for linear DNA molecules with substantial variation in length. For the shortest DNA (21 bp), the concentration of surfactant required to form stable complexes was the highest, while for longer nucleic acids the amount of surfactant is reduced for all studied systems. The long-chain DNA has exhibited the best complexing ability, and therefore, the lowest surfactant concentrations were sufficient to complex long-chain DNA molecules. Previous studies on the same DNA duplex showed that in a solution it assumes a rod-like structure with dimensions 7.2 nm in length and 2.2 nm in diameter [31]. This structure has all negative charges exposed, whereas longer DNA fragments possess higher conformational freedom and so are more likely to adopt coiled forms as a result of suppressing part of the native charge. On the other hand, the difference in complexation efficiency between systems containing 185 bp and 20 kbp DNA is almost inconsiderable. This suggests that there is a certain threshold of length in which the DNA is prone to form supercoiled forms upon interactions with surfactants. Comparing the complexation efficiency between the surfactants themselves in all studied formulations, there is a tendency that the smaller the polar group, the lower the surfactant concentration required for nucleic acid complexation. Two of the studied tricationic surfactants (TRI_N and TRI_IMI) were previously tested by us in mixtures without lipids with ds-DNA oligomers at lower DNA concentration [20]. Therefore, these two systems (TRI_N/DNA_21bp and TRI_IMI/DNA_ 21bp) served as reference in our study, and the obtained results were consistent.

Regarding properties, oligomeric surfactants with the increasing number of connected moieties show considerably improved properties. This was presented extensively for gemini (dimeric) surfactants in comparison to theirs monomeric counterparts [32,33]. This also extends to higher forms such as trimeric or tetrameric forms [34,35]. The downside of polymeric surfactants is increased toxic effects. Trimeric surfactants studied in this work are characterized by higher cytotoxicity than dimeric surfactants of similar composition already described in [15,17]. The *EC_50_* value (the concentration at which 50% of the cells died) for TRI_N surfactant was assessed as 15 ± 3 μM whereas its dimeric analogue was characterized by almost three times higher value (42.2 ± 0.2 μM) [15]. For TRI_IMI surfactant *EC_50_* = 24 ± 6 μM, and for its closest dimeric counterpart, this parameter was determined as 42.02 μM [17]. In all of these studies, MTT viability assay was used as a method to estimate surfactants cytotoxicity. We also decided to use an additional method in our research i.e., trypan blue staining [36]. Obtained cytotoxicity results were higher than calculated with dose-response curve based on MTT assay. Probably it is caused by the presence of active dehydrogenases under tested conditions, despite cell membrane disintegration. According to trypan blue staining, the studied surfactants increase the permeability of membranes or even locally destroy their structure causing uptake of the dye, and afterward, the cell’s self-repair mechanisms may remove the damage.

It can be assumed that the differences in *EC_50_* values for the tested trimeric surfactants and their dimeric counterparts result from differences in their structure and are related to an increase in the electric charge density of polymeric surfactants. The charge density increases the efficiency of binding with genetic material, but at the same time enhances cytotoxic effects [37]. Also for other series of dimeric and trimeric surfactants, it was shown that betaine trimeric surfactant have slightly higher aquatic toxicity than corresponding dimeric molecules [38], however strong differences were presented mostly depending on the hydrophobic tails length. Also, the type and length of the spacer group were pointed out as the main factor influencing oligomeric surfactant properties [34]. Therefore, to balance desired (transfection efficiency) and undesired features new oligomeric compounds can be synthesized and studied.

However, in this study, we decided to reduce the toxicity by adding only phospholipids naturally occurring in biological membranes such as DMPC, DPPC, and DOPE. The most cytotoxic surfactants are TRI_N and TRI _IMI, which simultaneously achieve the best complexation efficiencies, and the least toxic is TRI_BEN, which requires higher concentrations to form stable complexes with DNA. The addition of phospholipids lowers the cytotoxic effects vastly, e.g., change in *EC_50_* values from 15 μM to 762 μM for TRI_N surfactant in combination with DMPC. The phospholipid mixture that leads to the highest cytotoxicity reduction was the L2 mixture i.e., DMPC/DOPE in proportion 10:1. DMPC is a commonly occurring saturated-chain lipid frequently used to study lipid bilayers [39,40], and it is sufficient enough to reduce cytotoxicity of studied surfactants significantly. DOPE can form salt bridges with surfactants [41], therefore screening the positive charge of the system resulting in decreased cytotoxicity. Additionally, DOPE induces polymorphic behavior as it can induce lamellar-to-inverted-hexagonal phase transitions [42]. Interestingly, the L2 and L3 (DMPC/DPPC/DOPE) mixtures contained the same amount of DOPE, but the obtained *EC_50_* values differ significantly, with considerably worse results obtained for the latter. Based on molecular dynamic studies [40], combining phosphatidylcholine derivatives with different acyl chain lengths can significantly affect several bilayer properties (area per lipid, the bilayer thickness, the lateral diffusion). In our case, this phenomenon can explain the decrease of *EC_50_* values in systems where both DMPC and DPPC are present (L3). Probably due to the higher diffusion coefficient of DPPC, it may act as a destabilizing component in the studied systems.

Dong et al. [43] studied the influence of DOPE and the mixture of DOPE and DPPC on the systems based on gemini surfactants and DNA plasmid. Fluorescence correlation spectroscopy (FCS) results showed that systems based on surfactants and DNA were more heterogenous and formed complexes containing 5–35 plasmids, whereas the addition of phospholipid resulted in the formation of more homogenous complexes. The estimated amount of encapsulated plasmid molecules was 2–20. These findings correspond well with our AFM results that showed decreased dimensions of formed microstructures by multicomponent systems. In general, AFM images obtained for the reference sample—high-molecular DNA—were characterized by a coiled structure with an average height of 93 ± 6 nm. It should be noted that in higher ionic strength, this DNA should be presented as long, separated strands deposited on mica surface as it was previously shown [16] and described in other studies [44] when deposited on an appropriately prepared surface. In our case to compare dimensions of structures with amphiphilic molecules, we wanted to assess the size of DNA coiled aggregates. The addition of surfactant, such as in the case of systems with TRI_N surfactant, resulted in reduced dimensions of complexes, for example, the average height of formed spherical structures was estimated to be 48 ± 4 nm (for p/n = 5) and 58 ± 4 nm (for p/n = 8). This implies significant condensation of nucleic acid chains in the complex (for these charge ratios, unbounded DNA was not present) or decreased number of DNA molecules encapsulated. A similar effect was presented previously for dimeric imidazolium-derived surfactants with the same DNA, where the size of aggregates formed by surfactant/DNA was half the size compared to the coiled DNA structures [12]. The introduction of lipids to the system caused the formation of larger inhomogeneous and irregular aggregates, however, accompanied by spherical forms with further decreased average size. For example, the height of TRI_N/L2/DNA spherical aggregates was assessed as 26 ± 5 nm and for TRI_IMI/L3/DNA systems as 20 ± 6 nm. It is caused either by further condensation or more equalized DNA packaging. A similar stabilizing effect due to the incorporation of the phospholipids to complexes with gemini surfactants was postulated by Dong [43] but also due to the introduction of the PEG coverage to siRNA/lipoplexes [45].

The observed large structures are probably multilayered forms reported previously for other lipids/DNA complexes [46,47,48]. In addition, cryo-TEM studies of gemini amphiphilic compounds with DOPE [49] raveled the formation of two types of aggregates with a multilamellar pattern consisting of a series of bilayers with plasmid DNA sandwiched between them. These two types of lamellar packing are described as a cluster type and a Fingerprint type. This behavior may be the characteristic of studied samples themselves, but we cannot exclude interactions between formed structures while drying samples before AFM measurements, and as a result, combining smaller aggregates into conglomerates. Apart from the possible deformation of the soft sample by the cantilever tip, assessing information about only a small section of the sample and these uncontrollable interactions of amphiphilic structures can be considered as the main disadvantage of the AFM technique for studying lipoplexes. Nevertheless, Troiber et al. [50] made a comparison between techniques allowing particle size determination of polyplexes, such as dynamic light scattering (DLS), nanoparticle tracking analysis (NTA), FCS, and AFM; the latter is the best choice for heterogeneous samples. However, Dan et al. summarized the kinetic behavior of lipid/DNA systems, describing the flocculation of nucleic-acid carrying vesicles to form larger aggregates as a normal stage after the complexation [51]. This process gives rise to fractal-like structures, followed by local rearrangements until equilibrium is reached. Additionally, it was shown that the lipoplexes formed by a mixture of cationic phospholipids dioleoylethylphosphatidylcholine and dilauroylethylphosphatidylcholine are highly efficient transfection agents, whereas independently their efficiency was rather low [48]. It was suggested that the formed multilayered structures, promote multiple encounters between lipoplexes and cellular membranes, which is the feature required for efficient release of DNA from the complex, thus improving the transfection efficiency. It was also shown that these multilamellar structures, alternating with monolayers of ordered DNA cylinders, protect the nucleic acid thermally, while resulting only in a small degree of condensation and dehydration [47].

While the high degree of nucleic acid condensation is a desirable feature, maintaining its functional conformation is necessary to achieve therapeutic effects; therefore, we conducted CD studies (limited to two-components systems due to the high absorption of lipids—opaque samples). All studied nucleic acids were forming the native right-handed B-form, the most common under physiological conditions. The spectrum with the negative and positive peaks around 245–250 and 275–280 nm, respectively, represents the spectroscopic characteristic of B-form [16,18,19].

Summarizing the results of the CD studies of lipoplexes based on the surfactant TRI_N and TRI_IMI, as a result of interaction with DNA, the intensity of the spectrum decreased in comparison to the reference DNA spectrum, and the maximum of the positive band was shifted toward higher wavelengths. These changes have been attributed to the condensation of DNA and slight conformational changes due to dehydration in the vicinity of ribose rings of phosphate groups of DNA, as was previously reported for dimeric surfactants [12,52]. The collapse of the positive band used to be attributed to the B–C transition or forming modified B with 10.2 bp/turn instead of the usual 10.4 [12,53,54]; however, only in the case of DNA composed of 185 bp was the intensity of the negative band increased with the addition of surfactant.

TRI_N and TRI_IMI surfactants possess polar groups, which are relatively small and can be expected to directly interact with the phosphate groups of the DNA chain rather than penetrate the interior of the DNA helical structure, whereas in the case of TRI_BEN, observed spectral changes are more pronounced. For these systems, a rather fast collapse of the positive band is followed by the formation of a broad negative band in its place. It is visible for all studied nucleic acids; however, the intensity of the negative band revealed upon the addition of surfactant is the greater the smaller is the DNA molecule. This would imply the intense DNA condensation to the point of transition to the so-called *Ψ*-form [18,19]. *Ψ*-form was previously reported for several dimeric surfactants [16,55]. Moreover, TRI_BEN, according to the CD results, intensely condensates DNA also below the concentration required for complete nucleic acid complexation. This may imply that either condensation is easily reversible or other chiroptical features connected for example to surfactant polar head group rearrangements impact the spectra.

## 4. Materials and Methods

### 4.1. Cationic Trimeric Surfactants

**Figure 15:**
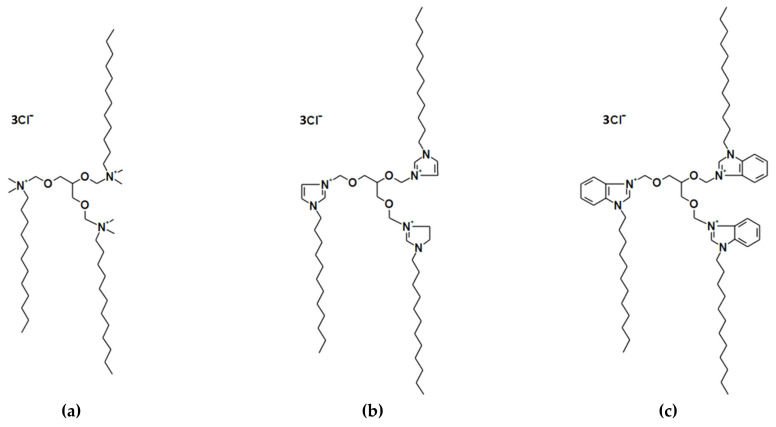
Chemical structures of studied trimeric surfactants with different polar group: (**a**) 1,2,3-propan-tri[oxymethyl-(*N*,*N*-dimethyldodecylammonium)] trichloride—TRI_N; (**b**) 1,2,3-propan-tri[oxymethyl-3-(1-dodecylimidazolium)] trichloride—TRI_IMI; and (**c**) 1,2,3-propan-tri[oxymethyl-3-(1-dodecylbenzimi-dazolium)] trichloride–TRI_BEN.

Cationic trimeric surfactants were prepared in the quaternization reaction between N,N-dimethyldodecylamine (Sigma-Aldrich, Saint Louis, MO, USA) or 1-dodecylimidazole or 1-dodecylbenzimidazole with 1,2,3-trichloromethoxypropane, as it was labeled in [56]. In addition, 1,2,3-trichloromethoxypropane was synthesized in the reaction described in [56]. 1-Dodecylimidazole and 1-dodecylbenzylimidazole were synthesized in the reaction described in [57]. Three cationic trimeric surfactants differ in type of polar groups were investigated. Namely, 1,2,3-propan-tri[oxymethyl-(*N*,*N*-dimethyldodecyl-ammonium)] trichloride denoted as TRI_N; 1,2,3-propan-tri[oxymethyl−3-(1-dodecylimidazolium)] trichloride denoted as TRI_IMI; and 1,2,3-propan-tri[oxymethyl-3-(1-dodecylbenzimidazolium)] trichloride denoted as TRI_BEN. The length of alkyl chains was the same for these surfactants, as well as chloride counter ions and the length of side chains, which consisted of saturated dodecyl chains. The chemical structures of the trimeric surfactants studied are presented in Figure 15.

### 4.2. Lipoplex Preparation

Studied lipoplexes are based on surfactant solution and lipid or lipids formulations, subsequently mixed with a nucleic acid solution; all components were prepared in a volume ratio of 1:2:1. Surfactants were dissolved in D_2_O and sonicated for 30 min at 50 °C. A stock solution of 21.4 mM was prepared, and each dilution step was followed by 10 min of sonication at 50 °C. Dilutions were prepared to obtain a specific p/n ratio. P/n ratio is defined as ratio of positive charge originating from surfactant molecules to the DNA’s negative charge and is calculated according to Equation (1).

(1) $$p/n\;=\frac{3\;\cdot \;C_{\mathrm{surf}}}{k\;\cdot \;C_{\mathrm{DNA}}}$$

Equation (1). Calculations of ratio of positive charge originating from surfactant molecules to the DNA’s negative charge. C_surf_—molar concentration of a surfactant; C_DNA_—molar concentration of DNA, k—the amount of positive electric charges per DNA molecule (e.g., for DNA 185 bp k = 370), and 3—the amount of negative electric charges per molecule of surfactant.

The range of tested values of p/n ratio was the following: 0.5, 0.75, 1, 2, 3, 4, 5, 6, 7, 8, 10, 12, 14, 16, 18, and 20. Therefore, the maximum concentration of surfactants in solution for p/n = 20 was 5.35 mM, and the minimal concentration for p/n = 0.5 was 0.13 mM.

The second component of studied lipoplexes was natural phospholipids and its mixtures. Namely, DMPC (1,2-dimyristoyl-*sn*-glycero-3-phosphocholine), DPPC (1,2-dipalmitoyl-*sn*-glycero-3-phosphocholine), and DOPE (1,2-dioleoyl-*sn*-glycero-3-phosphoethanolamine) were tested and purchased from Avanti Polar Lipids.

Three types of solutions have been investigated: one-component 10% DMPC solution—symbol L1, two-component solution of DMPC/DOPE (9%:1%)—symbol L2, and three-component solutions—DMPC/DPPC/DOPE (4.5%:4.5%:1%)—symbol L3. All solutions were prepared the same way: powdered samples were dissolved in D_2_O to obtain doubled concentration, and then samples were sonicated for 30 min at 50 °C, followed by incubation at −20 °C for 15 min. This procedure was repeated 10 times.

The tested complexes were combined with three types of nucleic acids, differing in length: high-molecular-weight DNA consisting of about 20 kbp (Sigma-Aldrich, Saint Louis, MO, USA), low-molecular-weight DNA—185 bp (Sigma-Aldrich, Saint Louis, MO, USA), and small double-stranded DNA (ds-DNA) oligomer—21 bp (FUTURE Synthesis Poland). The ds-DNA (21 bp) oligomer was previously tested by us at lower DNA concentrations (6–47 μM) with TRI_N and TRI_IMI surfactants [20]; therefore, we are using this system here as a reference.

Powdered nucleic acid samples were dissolved in 20 mM phosphate buffer (D_2_O, pH 6.8). To obtain specific p/n values, stock concentrations of nucleic acids solutions were: 76 μM for 21 bp DNA, 8.6 μM for 185 bp DNA, and 0.08 μM for 20 kbp DNA.

To prepare final multicomponent systems, mixtures of surfactants and phospholipids were sonicated for 10 min at 25 °C, and subsequently, the DNA solution was added, and the samples were incubated for 15 min at room temperature.

### 4.3. Electrophoretic Tests

Agarose gel electrophoresis in the presence of ethidium bromide (0.5 mg/mL) was applied to evaluate the concentration at which nucleic acid is bound by lipoplex but also to assess the degree of its release. To achieve good separation, 2% gels were prepared by dissolving an appropriate amount of agarose in TBE buffer (90 mM Tris base, 90 mM boric acid, and 2 mM disodium EDTA, pH 8). To the prepared lipoplex samples, 4 µL of loading buffer was added (0.25% bromophenol blue, 0.25% orange G, 40% glucose, and TBE buffer), and then 15 µL samples were placed in gel wells. As reference samples, a pure nucleic acid solution and a wide-range DNA marker (Sigma-Aldrich, Saint Louis, MO, USA) were used. Electrophoresis was carried out at 120 V for 45 min. To illuminate the electrophoresis gels, a standard UV transilluminator with 300 nm wavelength radiation was used.

To test the process of DNA release from the complexes, heparin solutions of various concentrations were prepared (from a stock concentration of 100 mg/mL), added to samples, and incubated for 30 min at room temperature, before adding the loading buffer and conducting electrophoretic tests.

### 4.4. Cytotoxicity Tests

#### 4.4.1. Cell Culture

Human cervical adenocarcinoma HeLa cell line was cultured in standard Dulbecco’s modified Eagle’s medium (DMEM) with 10% fetal bovine serum (FBS), 2 mM L-glutamine, and penicillin-streptomycin solution in the CytoGROW ™ GLP incubator (Sanyo, Wood Dale, IL, USA) at 37 °C and 5% CO_2_.

#### 4.4.2. Cytotoxicity Tests

We used the HeLa cell line to estimate surfactants cytotoxicity with MTT cell viability assay and the dye exclusion test (trypan blue). Day before experiment cells were passaged to a 96-well plate in 100 μL cell medium and cultured until 80% confluent. The level of confluence was monitored with a phase-contrast optical microscope (Axiovert 40 CFL, Zeiss, Jena, Germany). Cells were incubated with tested substances in the presence of clear DMEM medium in proportion 1:100 for one hour at culture conditions. At least 3 biological repetitions were performed, 2 technical repetitions each. Morphological changes in the HeLa cells after incubation were evaluated under ZEISS Observer.Z1.

#### 4.4.3. MTT Cell Viability Assay

After incubation with studied surfactants, 10 μL MTT—3-[4,5-dimethylthiazol-2-yl]-2,5-diphenyltetrazolium bromide (5 mg/mL MTT in PBS)—was added to each well and incubated for 3 h, as described previously. Then, the created formazan was dissolved in acidic isopropanol (0.04 M HCl in absolute isopropanol) in a 1:1 proportion. The result of the MTT cell viability assay was measured as absorbance at 570 nm using Tecan Infinite 200 pro microplate reader. The absorbance of the control sample (without studied substance) determined 100% viability. Vitality results of all tested substance concentrations are represented as the ratio of absorbance each sample to absorbance control sample multiply by 100%. Based on the dose-response curve, half maximal effective concentration (*EC_50_*) was determined.

#### 4.4.4. Trypan Blue Staining

After incubation with studied surfactants the medium was replaced with a PBS. Next, 0.4% trypan blue in PBS was added to the wells in 1:1 proportion and incubated for 5 min. Cells were observed in PBS. The staining effect was evaluated under optical microscope.

### 4.5. Circular Dichroism Spectroscopy

CD spectra were recorded to investigate structural changes of DNA occurred after adding surfactants. A J-815 JASCO spectropolarimeter and a 1 mm quartz cuvette were used. Spectra were recorded in the UV range (220–350 nm), at a scan rate of 100 nm/min with bandwidth of 1 nm and integration time of 2 s. To achieve a better signal-to-noise ratio, each spectrum was calculated as the average of five recorded scans. All measurements were carried out at room temperature (25 ± 1 °C). A background CD spectrum recorded a 5 mM phosphate buffer, which was subtracted from the sample spectra. All spectra were smoothed using the Savitzky-Golay function over 9 points. For analysis and presentation of obtained data, Spectra Manager II (Jasco, Tokyo, Japan) and Origin software were used. Due to high turbidity of samples with phospholipids, measurements were not possible.

### 4.6. Atomic Force Microscopy

The topographical study of the DNA samples and its complexes was conducted with Innova (Bruker, Billerica, MA, USA) atomic force microscope (AFM) in air environment. Immediately after drying process in about 23 °C, each sample prepared on mica substrate was probed in the AFM intermittent contact mode with the PPP-NCLR silicon cantilever (Nanosensors, Neuchatel, Switzerland). The data were collected with up to 1024 px resolution and analyzed using Gwyddion software [58].

## 5. Conclusions

As surfactant/lipids systems have great potential as drug carriers for gene therapy, the interaction of DNA (of various lengths) with cationic trimeric surfactants and lipids formulation based on mixtures of DMPC, DOPE, and DPPC has been investigated. These systems are characterized by high efficiency and low cytotoxicity. Moreover, the addition of lipids to the systems makes complexes more stable compared to surfactant/DNA complexes. The CD experiment shows that surfactants intensely condensate DNA in complexes, even causing the transition from B-form to so-called *Ψ*-DNA-form. AFM imaging shows formation of smaller structures for surfactant/lipid/DNA than for surfactant/DNA. Systems based on trimeric surfactants and mixtures of lipids (especially TRI_N and TRI_IMI) have all the necessary properties for characterizing good drug carriers and could be used as nonviral vectors with great success.

## 6. Patents

The patent application P.435691: Polańska, Ż., Pietralik-Molińska, Z., Skrzypczak, A., Kozak, M. “Multicomponent non-viral systems for binding and transfecting nucleic acids, the method of producing these systems and the application for introducing genetic material into living cells” was submitted on 15.10.2020 to the Patent Office of the Republic of Poland

## Figures and Tables

**Table 1:** The efficiency of the binding of the nucleic acid by studied systems.

DNA.	Lipids/Surfactant	TRI_N	TRI_IMI	TRI_BEN
**20 kbp**	-	4	5	14
	L1	2	2	4
	L2	1	1	2
	L3	0.75	2	4
**185 bp**	-	4	5	16
	L1	2	2	14
	L2	3	3	10
	L3	3	2	10
**21 bp**	-	8	8	18
	L1	6	6	16
	L2	5	6	16
	L3	6	6	16

**Table 2:** Results of heparin induced release of the high-molecular-weight DNA with studied amphiphilic complexes.

Complex.		Heparin Concentration during Release (µg/mL)			
Surfactant	p/n	-	L1	L2	L3
TRI_N	5	2.5	10	10	10
	10	2.5	10	10	10
	20	10	25	25	25
TRI_IMI	5	2.5	10	10	10
	10	2.5	10	10	10
	20	10	25	25	10
TRI_BEN	10	-	2.5	2.5	2.5
	20	1	10	10	10

**Table 3:** Results of half maximal effective concentration—*EC_50_*—for studied lipoplexes.

Surfactant	Lipids	*EC_50_* (µM)
**TRI_N**	-	15 ± 3
	L1	762 ± 12
	L2	856 ± 24
	L3	302 ± 6
**TRI_IMI**	-	24 ± 6
	L1	723 ± 15
	L2	962 ± 18
	L3	578 ± 12
**TRI_BEN**	-	60 ± 12
	L1	526 ± 21
	L2	648 ± 24
	L3	294 ± 15

**Table 4:** Summary of trypan blue staining results for studied surfactants and surfactants/lipids mixtures. x—used when the most cells were blue or dark blue.

System	Surfactant Concentration (µM)							
	0	4	30	60	100	500	1000	2000
TRI_N		x	x	x	x	x	x	x
TRI_IMI		x	x	x	x	x	x	x
TRI_BEN			x	x	x	x	x	x
Surfactants and lipids					x	x	x	x

## Data Availability

The data presented in this study are available on request from the corresponding author.
